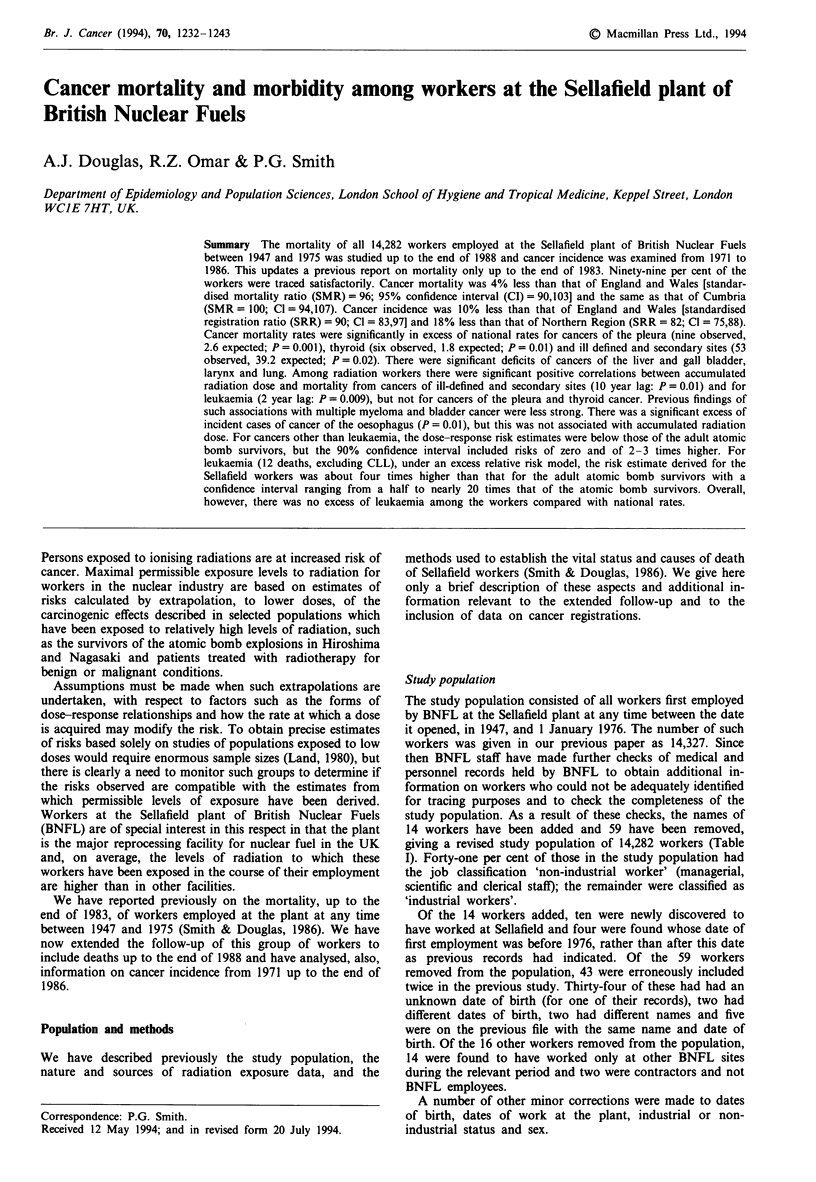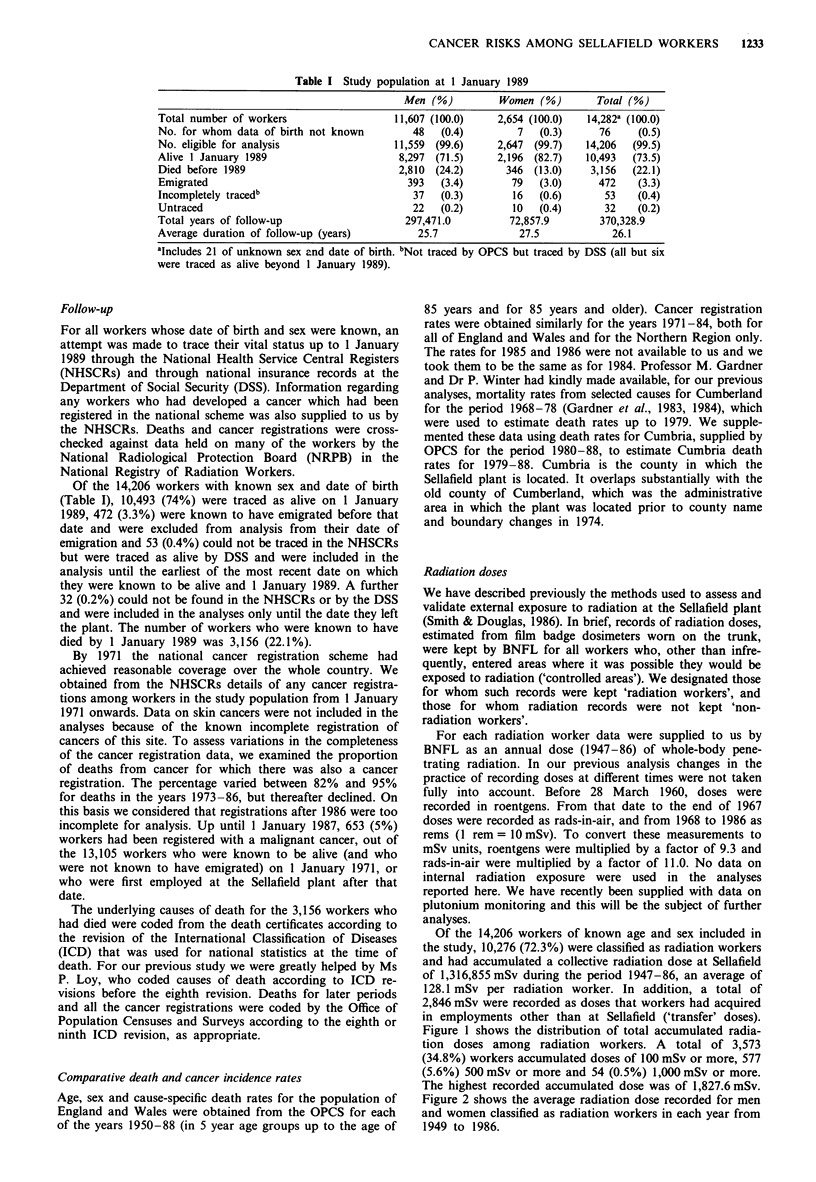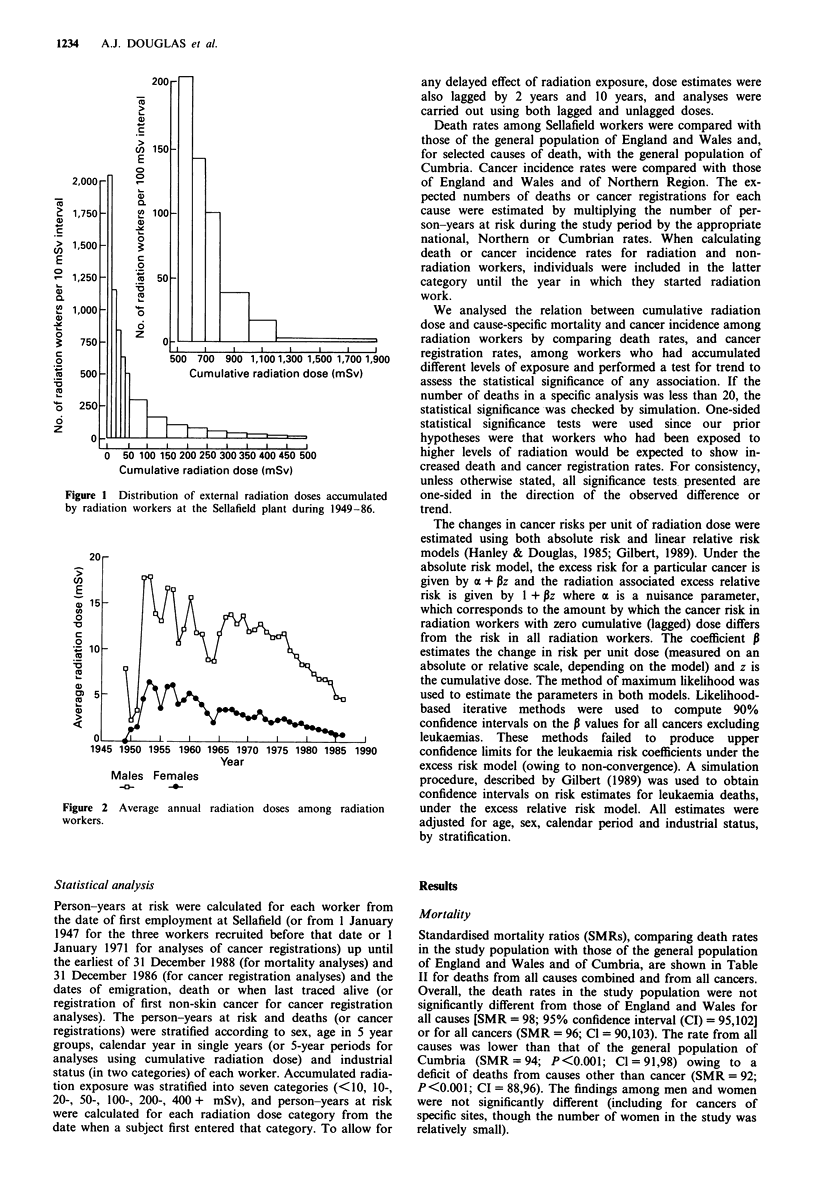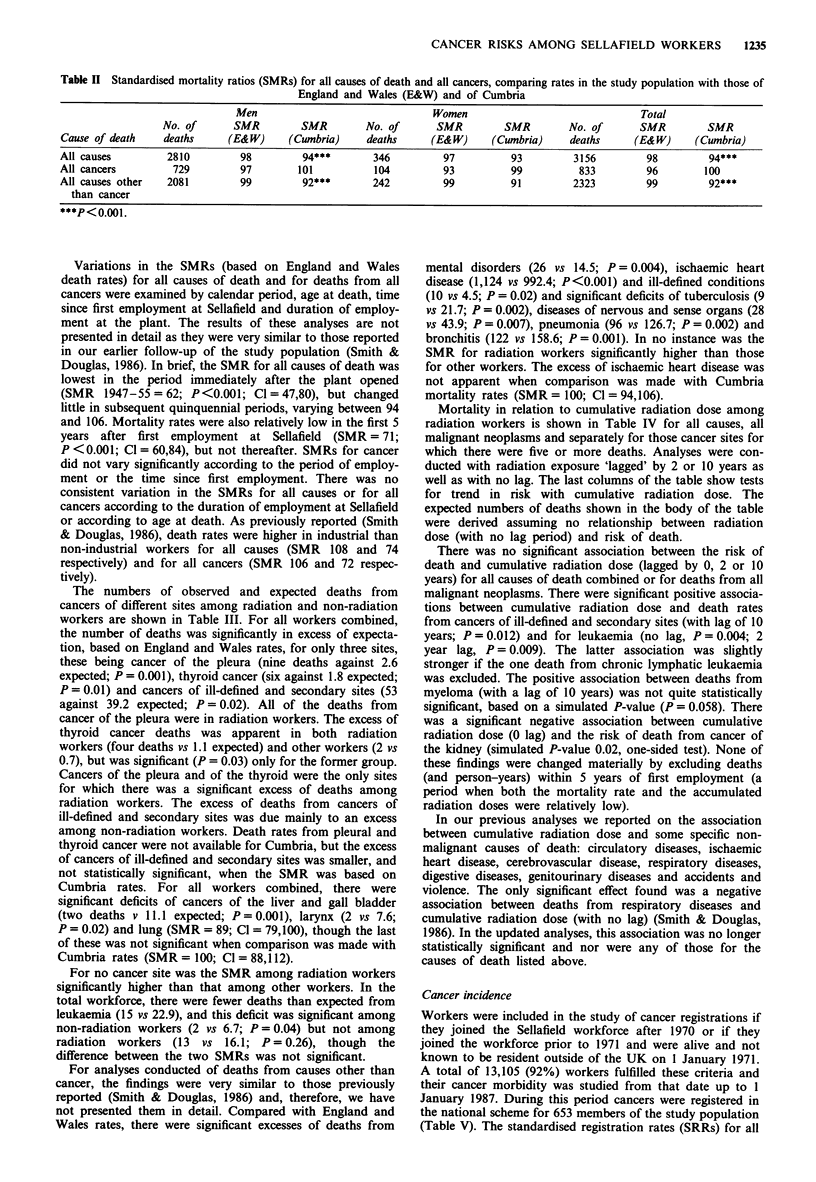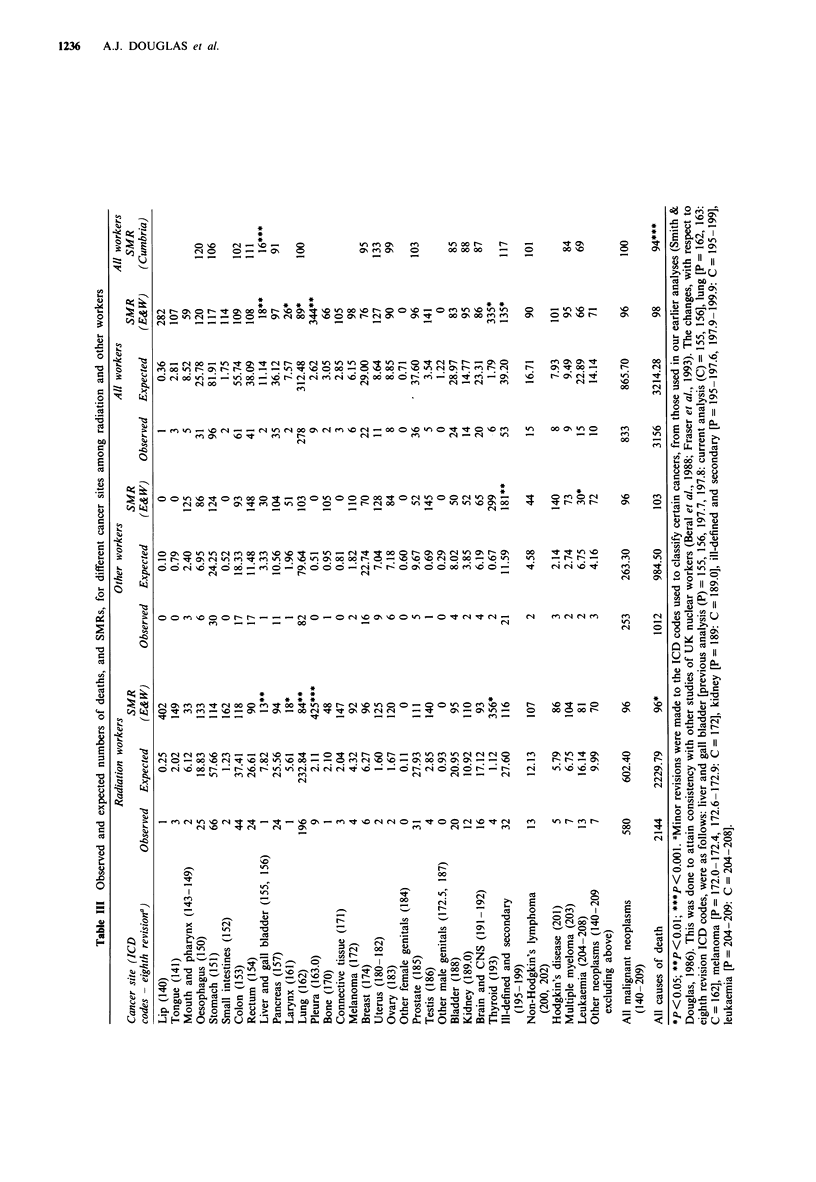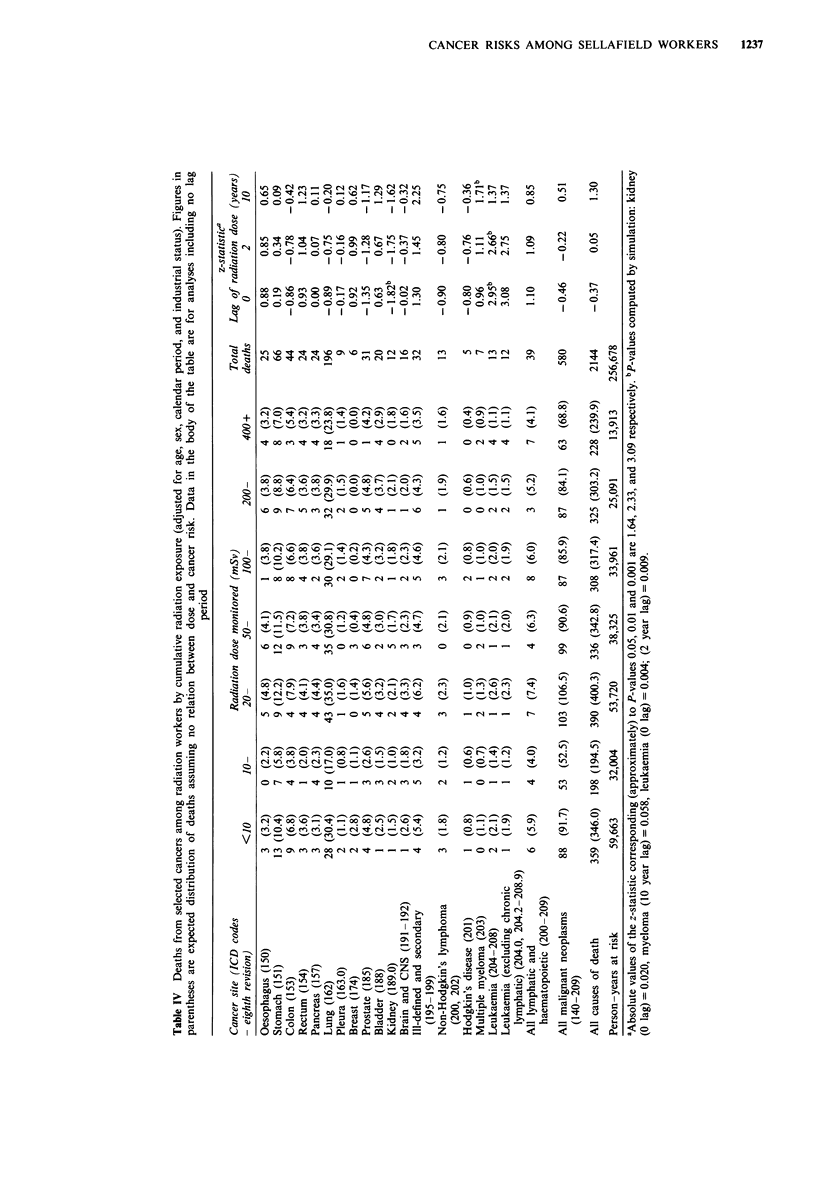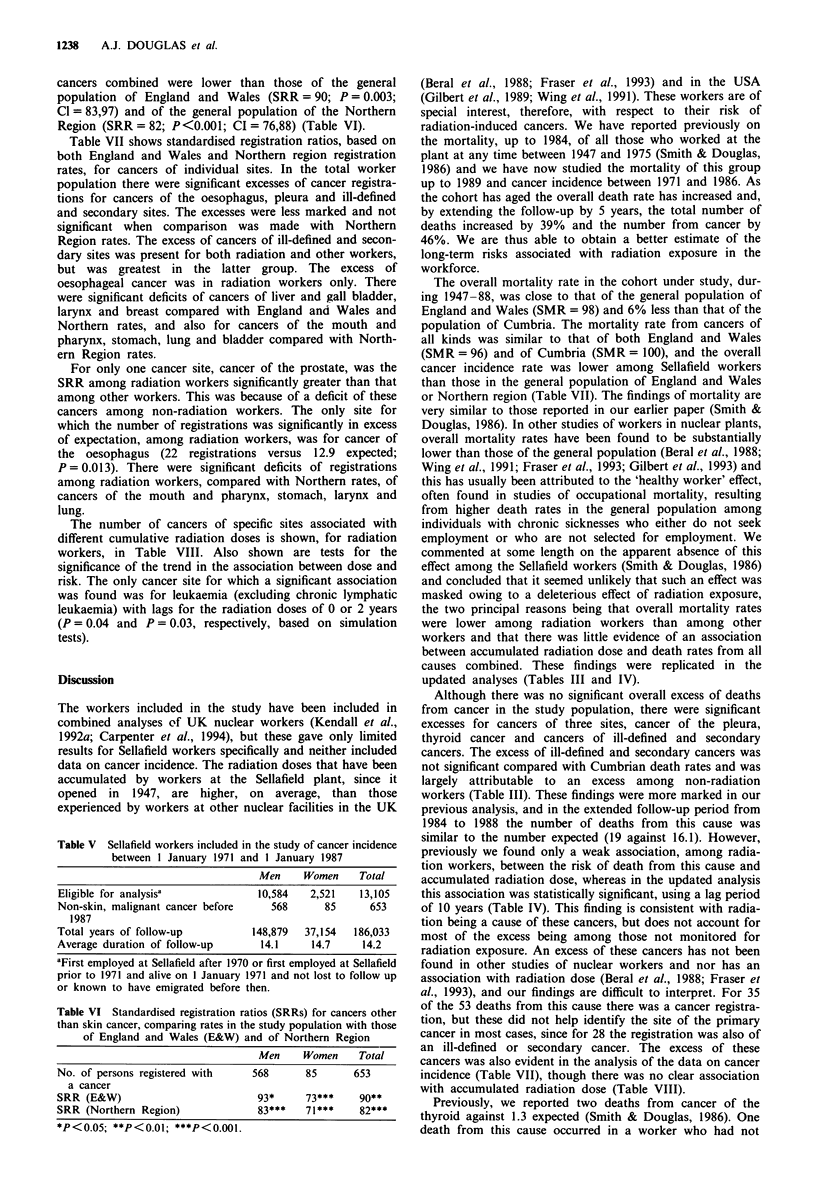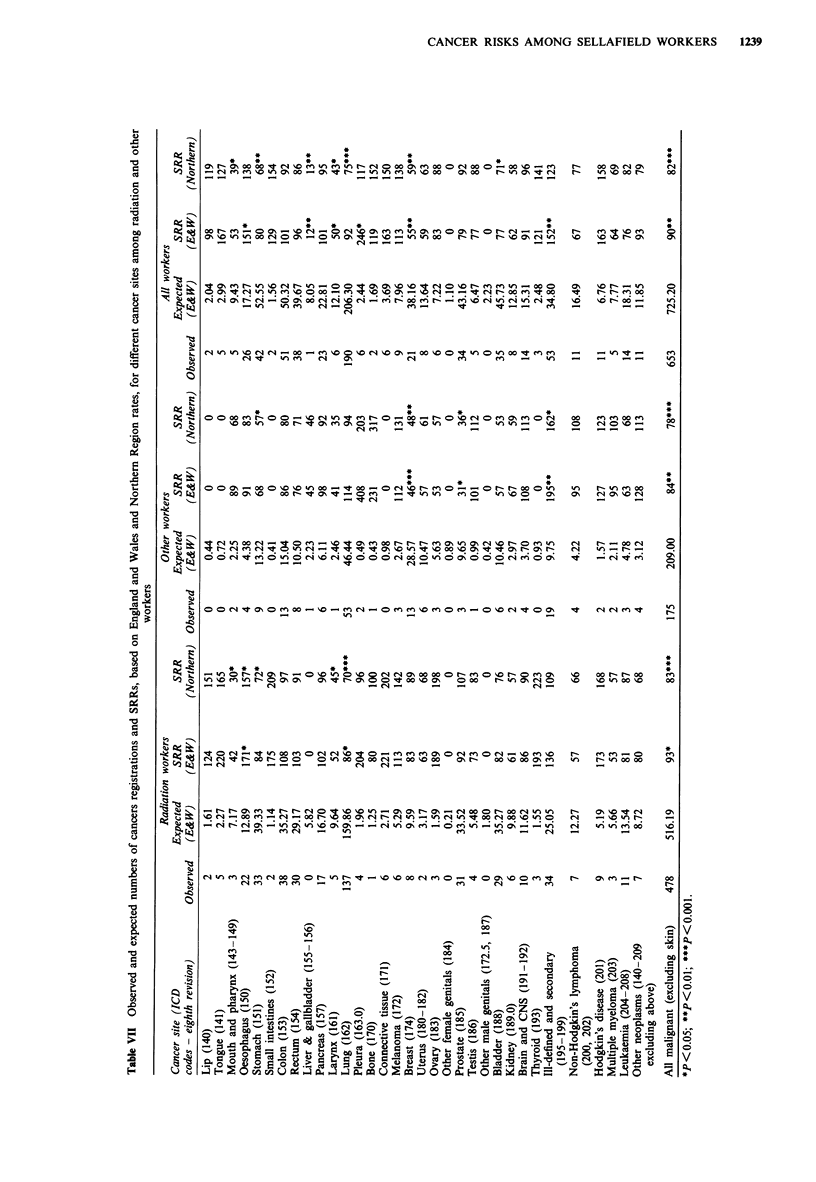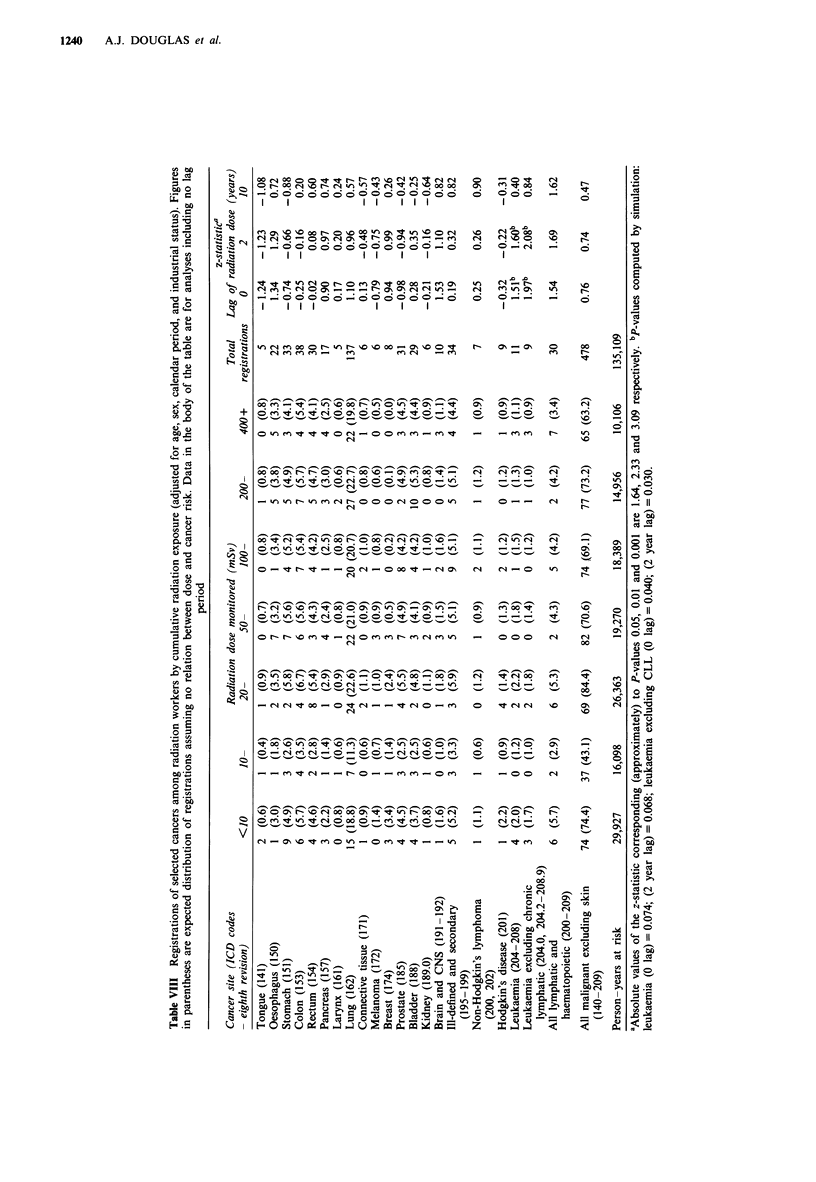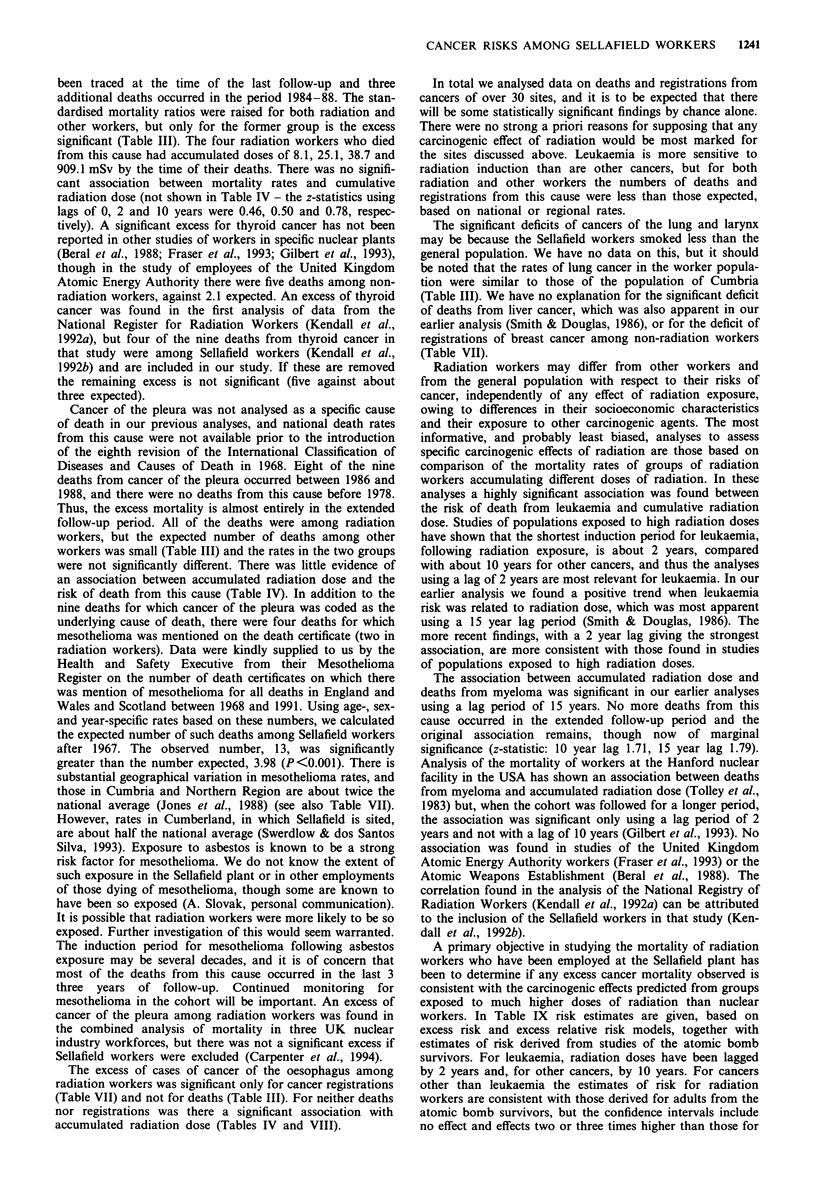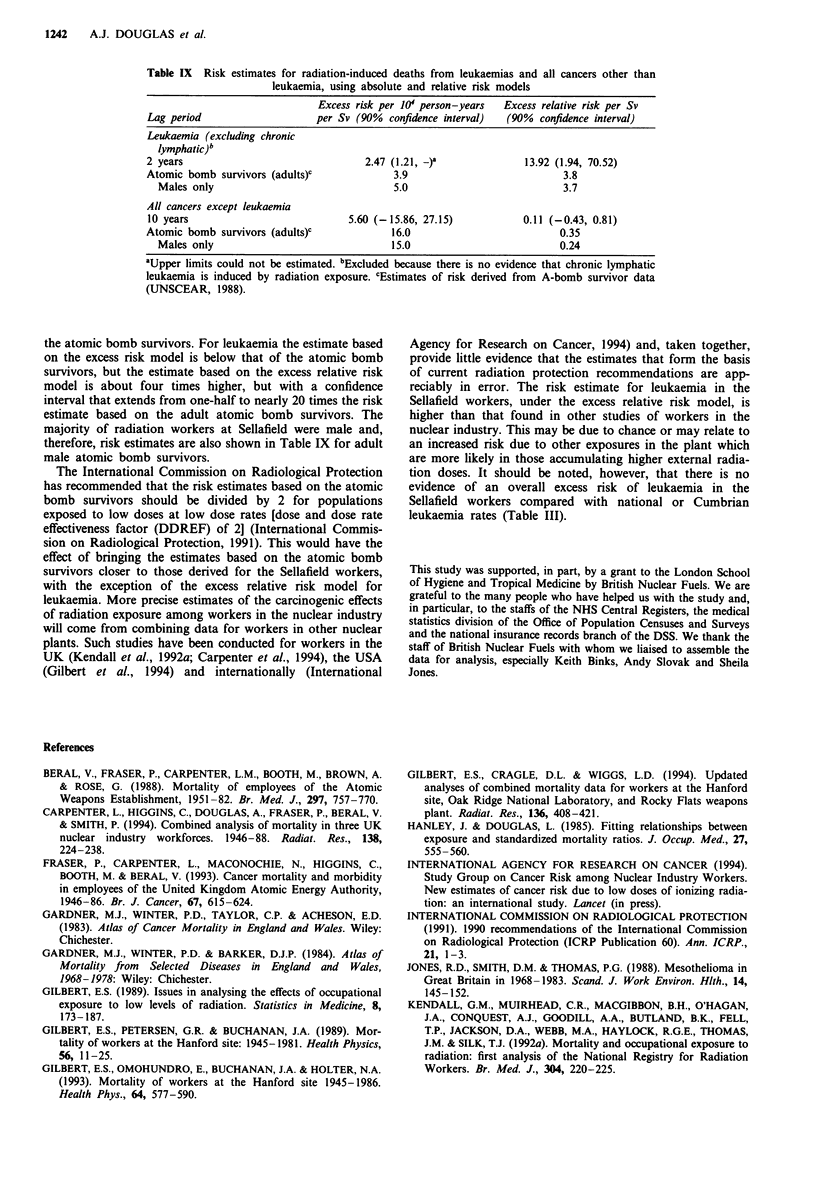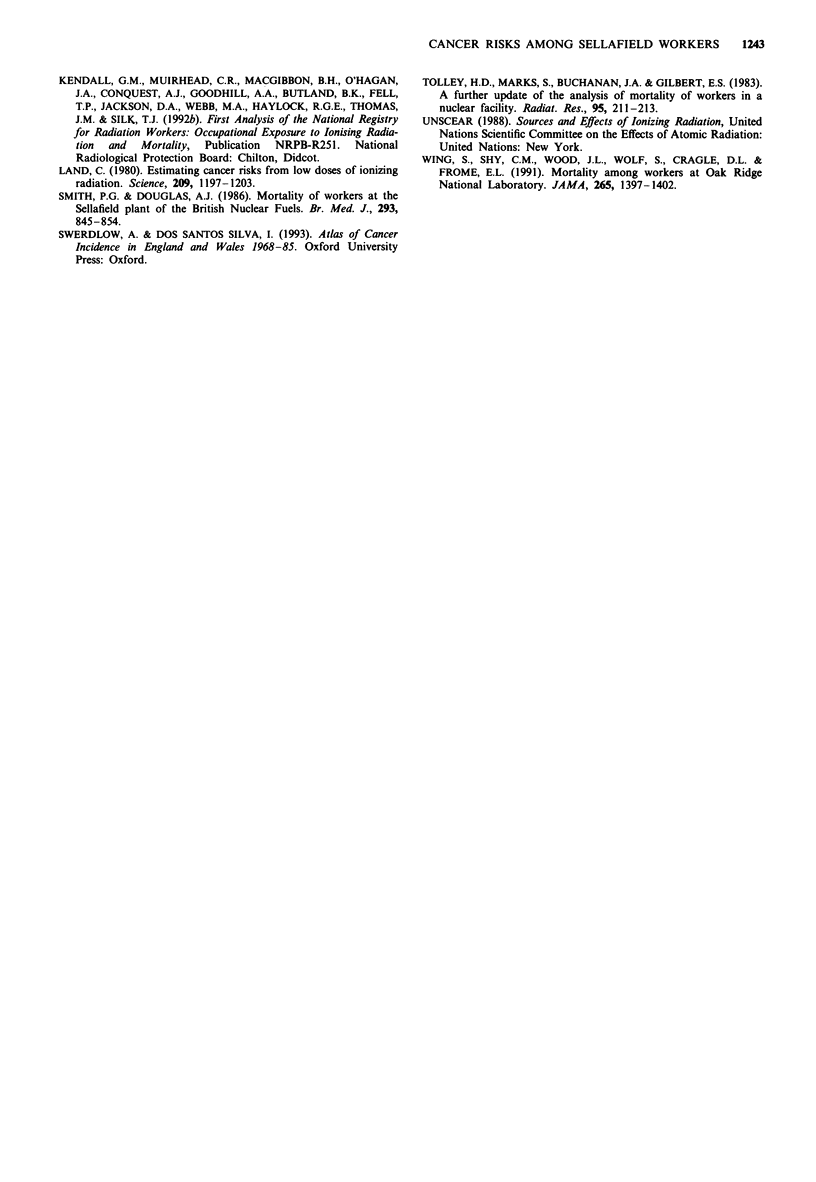# Cancer mortality and morbidity among workers at the Sellafield plant of British Nuclear Fuels.

**DOI:** 10.1038/bjc.1994.479

**Published:** 1994-12

**Authors:** A. J. Douglas, R. Z. Omar, P. G. Smith

**Affiliations:** Department of Epidemiology and Population Sciences, London School of Hygiene and Tropical Medicine, UK.

## Abstract

The mortality of all 14,282 workers employed at the Sellafield plant of British Nuclear Fuels between 1947 and 1975 was studied up to the end of 1988 and cancer incidence was examined from 1971 to 1986. This updates a previous report on mortality only up to the end of 1983. Ninety-nine per cent of the workers were traced satisfactorily. Cancer mortality was 4% less than that of England and Wales [standardised mortality ratio (SMR) = 96; 95% confidence interval (CI) = 90,103] and the same as that of Cumbria (SMR = 100: Cl = 94,107). Cancer incidence was 10% less than that of England and Wales [standardised registration ratio (SRR) = 90; Cl = 83.97] and 18% less than that of Northern Region (SRR = 82; Cl = 75.88). Cancer mortality rates were significantly in excess of national rates for cancers of the pleura (nine observed, 2.6 expected; P = 0.001), thyroid (six observed, 1.8 expected; P = 0.01) and ill defined and secondary sites (53 observed, 39.2 expected; P = 0.02). There were significant deficits of cancers of the liver and gall bladder, larynx and lung. Among radiation workers there were significant positive correlations between accumulated radiation dose and mortality from cancers of ill-defined and secondary sites (10 year lag: P = 0.01) and for leukaemia (2 year lag: P = 0.009), but not for cancers of the pleura and thyroid cancer. Previous findings of such associations with multiple myeloma and bladder cancer were less strong. There was a significant excess of incident cases of cancer of the oesophagus (P = 0.01), but this was not associated with accumulated radiation dose. For cancers other than leukaemia, the dose-response risk estimates were below those of the adult atomic bomb survivors, but the 90% confidence interval included risks of zero and of 2-3 times higher. For leukaemia (12 deaths, excluding CLL), under an excess relative risk model, the risk estimate derived for the Sellafield workers was about four times higher than that for the adult atomic bomb survivors with a confidence interval ranging from a half to nearly 20 times that of the atomic bomb survivors. Overall, however, there was no excess of leukaemia among the workers compared with national rates.


Br. J. Cancer (1994), 70, 1232–1243 © Macmillan Press Ltd., 1994

### Cancer mortality and morbidity among workers at the Sellafield plant of British Nuclear Fuels

A.J. Douglas, R.Z. Omar & P.G. Smith

*Department of Epidemiology and Population Sciences, London School of Hygiene and Tropical Medicine, Keppel Street, London WC1E 7HT, UK.*

**Summary** The mortality of all 14,282 workers employed at the Sellafield plant of British Nuclear Fuels between 1947 and 1975 was studied up to the end of 1988 and cancer incidence was examined from 1971 to 1986. This updates a previous report on mortality only up to the end of 1983. Ninety-nine per cent of the workers were traced satisfactorily. Cancer mortality was 4% less than that of England and Wales [standardised mortality ratio (SMR) = 96; 95% confidence interval (CI) = 90,103] and the same as that of Cumbria (SMR = 100; CI = 94,107). Cancer incidence was 10% less than that of England and Wales [standardised registration ratio (SRR) = 90; CI = 83,97] and 18% less than that of Northern Region (SRR = 82; CI = 75,88). Cancer mortality rates were significantly in excess of national rates for cancers of the pleura (nine observed, 2.6 expected; $P = 0.001$), thyroid (six observed, 1.8 expected; $P = 0.01$) and ill defined and secondary sites (53 observed, 39.2 expected; $P = 0.02$). There were significant deficits of cancers of the liver and gall bladder, larynx and lung. Among radiation workers there were significant positive correlations between accumulated radiation dose and mortality from cancers of ill-defined and secondary sites (10 year lag: $P = 0.01$) and for leukaemia (2 year lag: $P = 0.009$), but not for cancers of the pleura and thyroid cancer. Previous findings of such associations with multiple myeloma and bladder cancer were less strong. There was a significant excess of incident cases of cancer of the oesophagus ($P = 0.01$), but this was not associated with accumulated radiation dose. For cancers other than leukaemia, the dose–response risk estimates were below those of the adult atomic bomb survivors, but the 90% confidence interval included risks of zero and of 2–3 times higher. For leukaemia (12 deaths, excluding CLL), under an excess relative risk model, the risk estimate derived for the Sellafield workers was about four times higher than that for the adult atomic bomb survivors with a confidence interval ranging from a half to nearly 20 times that of the atomic bomb survivors. Overall, however, there was no excess of leukaemia among the workers compared with national rates.

Persons exposed to ionising radiations are at increased risk of cancer. Maximal permissible exposure levels to radiation for workers in the nuclear industry are based on estimates of risks calculated by extrapolation, to lower doses, of the carcinogenic effects described in selected populations which have been exposed to relatively high levels of radiation, such as the survivors of the atomic bomb explosions in Hiroshima and Nagasaki and patients treated with radiotherapy for benign or malignant conditions.

Assumptions must be made when such extrapolations are undertaken, with respect to factors such as the forms of dose–response relationships and how the rate at which a dose is acquired may modify the risk. To obtain precise estimates of risks based solely on studies of populations exposed to low doses would require enormous sample sizes (Land, 1980), but there is clearly a need to monitor such groups to determine if the risks observed are compatible with the estimates from which permissible levels of exposure have been derived. Workers at the Sellafield plant of British Nuclear Fuels (BNFL) are of special interest in this respect in that the plant is the major reprocessing facility for nuclear fuel in the UK and, on average, the levels of radiation to which these workers have been exposed in the course of their employment are higher than in other facilities.

We have reported previously on the mortality, up to the end of 1983, of workers employed at the plant at any time between 1947 and 1975 (Smith & Douglas, 1986). We have now extended the follow-up of this group of workers to include deaths up to the end of 1988 and have analysed, also, information on cancer incidence from 1971 up to the end of 1986.

### Population and methods

We have described previously the study population, the nature and sources of radiation exposure data, and the methods used to establish the vital status and causes of death of Sellafield workers (Smith & Douglas, 1986). We give here only a brief description of these aspects and additional information relevant to the extended follow-up and to the inclusion of data on cancer registrations.

### *Study population*

The study population consisted of all workers first employed by BNFL at the Sellafield plant at any time between the date it opened, in 1947, and 1 January 1976. The number of such workers was given in our previous paper as 14,327. Since then BNFL staff have made further checks of medical and personnel records held by BNFL to obtain additional information on workers who could not be adequately identified for tracing purposes and to check the completeness of the study population. As a result of these checks, the names of 14 workers have been added and 59 have been removed, giving a revised study population of 14,282 workers (Table I). Forty-one per cent of those in the study population had the job classification 'non-industrial worker' (managerial, scientific and clerical staff); the remainder were classified as 'industrial workers'.

Of the 14 workers added, ten were newly discovered to have worked at Sellafield and four were found whose date of first employment was before 1976, rather than after this date as previous records had indicated. Of the 59 workers removed from the population, 43 were erroneously included twice in the previous study. Thirty-four of these had had an unknown date of birth (for one of their records), two had different dates of birth, two had different names and five were on the previous file with the same name and date of birth. Of the 16 other workers removed from the population, 14 were found to have worked only at other BNFL sites during the relevant period and two were contractors and not BNFL employees.

A number of other minor corrections were made to dates of birth, dates of work at the plant, industrial or non-industrial status and sex.

Correspondence: P.G. Smith.
Received 12 May 1994; and in revised form 20 July 1994.

**Table I** Study population at 1 January 1989

| | *Men (%)* | *Women (%)* | *Total (%)* |
|---|---|---|---|
| Total number of workers | 11,607 (100.0) | 2,654 (100.0) | 14,282[a] (100.0) |
| No. for whom data of birth not known | 48 (0.4) | 7 (0.3) | 76 (0.5) |
| No. eligible for analysis | 11,559 (99.6) | 2,647 (99.7) | 14,206 (99.5) |
| Alive 1 January 1989 | 8,297 (71.5) | 2,196 (82.7) | 10,493 (73.5) |
| Died before 1989 | 2,810 (24.2) | 346 (13.0) | 3,156 (22.1) |
| Emigrated | 393 (3.4) | 79 (3.0) | 472 (3.3) |
| Incompletely traced[b] | 37 (0.3) | 16 (0.6) | 53 (0.4) |
| Untraced | 22 (0.2) | 10 (0.4) | 32 (0.2) |
| Total years of follow-up | 297,471.0 | 72,857.9 | 370,328.9 |
| Average duration of follow-up (years) | 25.7 | 27.5 | 26.1 |

[a]Includes 21 of unknown sex and date of birth. [b]Not traced by OPCS but traced by DSS (all but six were traced as alive beyond 1 January 1989).

*Follow-up*

For all workers whose date of birth and sex were known, an attempt was made to trace their vital status up to 1 January 1989 through the National Health Service Central Registers (NHSCRs) and through national insurance records at the Department of Social Security (DSS). Information regarding any workers who had developed a cancer which had been registered in the national scheme was also supplied to us by the NHSCRs. Deaths and cancer registrations were cross-checked against data held on many of the workers by the National Radiological Protection Board (NRPB) in the National Registry of Radiation Workers.

Of the 14,206 workers with known sex and date of birth (Table I), 10,493 (74%) were traced as alive on 1 January 1989, 472 (3.3%) were known to have emigrated before that date and were excluded from analysis from their date of emigration and 53 (0.4%) could not be traced in the NHSCRs but were traced as alive by DSS and were included in the analysis until the earliest of the most recent date on which they were known to be alive and 1 January 1989. A further 32 (0.2%) could not be found in the NHSCRs or by the DSS and were included in the analyses only until the date they left the plant. The number of workers who were known to have died by 1 January 1989 was 3,156 (22.1%).

By 1971 the national cancer registration scheme had achieved reasonable coverage over the whole country. We obtained from the NHSCRs details of any cancer registrations among workers in the study population from 1 January 1971 onwards. Data on skin cancers were not included in the analyses because of the known incomplete registration of cancers of this site. To assess variations in the completeness of the cancer registration data, we examined the proportion of deaths from cancer for which there was also a cancer registration. The percentage varied between 82% and 95% for deaths in the years 1973–86, but thereafter declined. On this basis we considered that registrations after 1986 were too incomplete for analysis. Up until 1 January 1987, 653 (5%) workers had been registered with a malignant cancer, out of the 13,105 workers who were known to be alive (and who were not known to have emigrated) on 1 January 1971, or who were first employed at the Sellafield plant after that date.

The underlying causes of death for the 3,156 workers who had died were coded from the death certificates according to the revision of the International Classification of Diseases (ICD) that was used for national statistics at the time of death. For our previous study we were greatly helped by Ms P. Loy, who coded causes of death according to ICD revisions before the eighth revision. Deaths for later periods and all the cancer registrations were coded by the Office of Population Censuses and Surveys according to the eighth or ninth ICD revision, as appropriate.

*Comparative death and cancer incidence rates*

Age, sex and cause-specific death rates for the population of England and Wales were obtained from the OPCS for each of the years 1950–88 (in 5 year age groups up to the age of 85 years and for 85 years and older). Cancer registration rates were obtained similarly for the years 1971–84, both for all of England and Wales and for the Northern Region only. The rates for 1985 and 1986 were not available to us and we took them to be the same as for 1984. Professor M. Gardner and Dr P. Winter had kindly made available, for our previous analyses, mortality rates from selected causes for Cumberland for the period 1968–78 (Gardner *et al.*, 1983, 1984), which were used to estimate death rates up to 1979. We supplemented these data using death rates for Cumbria, supplied by OPCS for the period 1980–88, to estimate Cumbria death rates for 1979–88. Cumbria is the county in which the Sellafield plant is located. It overlaps substantially with the old county of Cumberland, which was the administrative area in which the plant was located prior to county name and boundary changes in 1974.

*Radiation doses*

We have described previously the methods used to assess and validate external exposure to radiation at the Sellafield plant (Smith & Douglas, 1986). In brief, records of radiation doses, estimated from film badge dosimeters worn on the trunk, were kept by BNFL for all workers who, other than infrequently, entered areas where it was possible they would be exposed to radiation ('controlled areas'). We designated those for whom such records were kept 'radiation workers', and those for whom radiation records were not kept 'non-radiation workers'.

For each radiation worker data were supplied to us by BNFL as an annual dose (1947–86) of whole-body penetrating radiation. In our previous analysis changes in the practice of recording doses at different times were not taken fully into account. Before 28 March 1960, doses were recorded in roentgens. From that date to the end of 1967 doses were recorded as rads-in-air, and from 1968 to 1986 as rems (1 rem = 10 mSv). To convert these measurements to mSv units, roentgens were multiplied by a factor of 9.3 and rads-in-air were multiplied by a factor of 11.0. No data on internal radiation exposure were used in the analyses reported here. We have recently been supplied with data on plutonium monitoring and this will be the subject of further analyses.

Of the 14,206 workers of known age and sex included in the study, 10,276 (72.3%) were classified as radiation workers and had accumulated a collective radiation dose at Sellafield of 1,316,855 mSv during the period 1947–86, an average of 128.1 mSv per radiation worker. In addition, a total of 2,846 mSv were recorded as doses that workers had acquired in employments other than at Sellafield ('transfer' doses). Figure 1 shows the distribution of total accumulated radiation doses among radiation workers. A total of 3,573 (34.8%) workers accumulated doses of 100 mSv or more, 577 (5.6%) 500 mSv or more and 54 (0.5%) 1,000 mSv or more. The highest recorded accumulated dose was of 1,827.6 mSv. Figure 2 shows the average radiation dose recorded for men and women classified as radiation workers in each year from 1949 to 1986.

**Figure 1** Distribution of external radiation doses accumulated by radiation workers at the Sellafield plant during 1949–86.

**Figure 2** Average annual radiation doses among radiation workers.

*Statistical analysis*

Person–years at risk were calculated for each worker from the date of first employment at Sellafield (or from 1 January 1947 for the three workers recruited before that date or 1 January 1971 for analyses of cancer registrations) up until the earliest of 31 December 1988 (for mortality analyses) and 31 December 1986 (for cancer registration analyses) and the dates of emigration, death or when last traced alive (or registration of first non-skin cancer for cancer registration analyses). The person–years at risk and deaths (or cancer registrations) were stratified according to sex, age in 5 year groups, calendar year in single years (or 5-year periods for analyses using cumulative radiation dose) and industrial status (in two categories) of each worker. Accumulated radiation exposure was stratified into seven categories (<10, 10-, 20-, 50-, 100-, 200-, 400 + mSv), and person–years at risk were calculated for each radiation dose category from the date when a subject first entered that category. To allow for any delayed effect of radiation exposure, dose estimates were also lagged by 2 years and 10 years, and analyses were carried out using both lagged and unlagged doses.

Death rates among Sellafield workers were compared with those of the general population of England and Wales and, for selected causes of death, with the general population of Cumbria. Cancer incidence rates were compared with those of England and Wales and of Northern Region. The expected numbers of deaths or cancer registrations for each cause were estimated by multiplying the number of person–years at risk during the study period by the appropriate national, Northern or Cumbrian rates. When calculating death or cancer incidence rates for radiation and non-radiation workers, individuals were included in the latter category until the year in which they started radiation work.

We analysed the relation between cumulative radiation dose and cause-specific mortality and cancer incidence among radiation workers by comparing death rates, and cancer registration rates, among workers who had accumulated different levels of exposure and performed a test for trend to assess the statistical significance of any association. If the number of deaths in a specific analysis was less than 20, the statistical significance was checked by simulation. One-sided statistical significance tests were used since our prior hypotheses were that workers who had been exposed to higher levels of radiation would be expected to show increased death and cancer registration rates. For consistency, unless otherwise stated, all significance tests presented are one-sided in the direction of the observed difference or trend.

The changes in cancer risks per unit of radiation dose were estimated using both absolute risk and linear relative risk models (Hanley & Douglas, 1985; Gilbert, 1989). Under the absolute risk model, the excess risk for a particular cancer is given by $\alpha + \beta z$ and the radiation associated excess relative risk is given by $1 + \beta z$ where $\alpha$ is a nuisance parameter, which corresponds to the amount by which the cancer risk in radiation workers with zero cumulative (lagged) dose differs from the risk in all radiation workers. The coefficient $\beta$ estimates the change in risk per unit dose (measured on an absolute or relative scale, depending on the model) and $z$ is the cumulative dose. The method of maximum likelihood was used to estimate the parameters in both models. Likelihood-based iterative methods were used to compute 90% confidence intervals on the $\beta$ values for all cancers excluding leukaemias. These methods failed to produce upper confidence limits for the leukaemia risk coefficients under the excess risk model (owing to non-convergence). A simulation procedure, described by Gilbert (1989) was used to obtain confidence intervals on risk estimates for leukaemia deaths, under the excess relative risk model. All estimates were adjusted for age, sex, calendar period and industrial status, by stratification.

### Results

*Mortality*

Standardised mortality ratios (SMRs), comparing death rates in the study population with those of the general population of England and Wales and of Cumbria, are shown in Table II for deaths from all causes combined and from all cancers. Overall, the death rates in the study population were not significantly different from those of England and Wales for all causes [SMR = 98; 95% confidence interval (CI) = 95,102] or for all cancers (SMR = 96; Cl = 90,103). The rate from all causes was lower than that of the general population of Cumbria (SMR = 94; $P<0.001$; Cl = 91,98) owing to a deficit of deaths from causes other than cancer (SMR = 92; $P<0.001$; CI = 88,96). The findings among men and women were not significantly different (including for cancers of specific sites, though the number of women in the study was relatively small).

**Table II** Standardised mortality ratios (SMRs) for all causes of death and all cancers, comparing rates in the study population with those of England and Wales (E&W) and of Cumbria

| Cause of death | Men No. of deaths | Men SMR (E&W) | Men SMR (Cumbria) | Women No. of deaths | Women SMR (E&W) | Women SMR (Cumbria) | Total No. of deaths | Total SMR (E&W) | Total SMR (Cumbria) |
|---|---|---|---|---|---|---|---|---|---|
| All causes | 2810 | 98 | 94*** | 346 | 97 | 93 | 3156 | 98 | 94*** |
| All cancers | 729 | 97 | 101 | 104 | 93 | 99 | 833 | 96 | 100 |
| All causes other than cancer | 2081 | 99 | 92*** | 242 | 99 | 91 | 2323 | 99 | 92*** |

***$P<0.001$.

Variations in the SMRs (based on England and Wales death rates) for all causes of death and for deaths from all cancers were examined by calendar period, age at death, time since first employment at Sellafield and duration of employment at the plant. The results of these analyses are not presented in detail as they were very similar to those reported in our earlier follow-up of the study population (Smith & Douglas, 1986). In brief, the SMR for all causes of death was lowest in the period immediately after the plant opened (SMR 1947-55 = 62; $P<0.001$; Cl = 47,80), but changed little in subsequent quinquennial periods, varying between 94 and 106. Mortality rates were also relatively low in the first 5 years after first employment at Sellafield (SMR = 71; $P<0.001$; Cl = 60,84), but not thereafter. SMRs for cancer did not vary significantly according to the period of employment or the time since first employment. There was no consistent variation in the SMRs for all causes or for all cancers according to the duration of employment at Sellafield or according to age at death. As previously reported (Smith & Douglas, 1986), death rates were higher in industrial than non-industrial workers for all causes (SMR 108 and 74 respectively) and for all cancers (SMR 106 and 72 respectively).

The numbers of observed and expected deaths from cancers of different sites among radiation and non-radiation workers are shown in Table III. For all workers combined, the number of deaths was significantly in excess of expectation, based on England and Wales rates, for only three sites, these being cancer of the pleura (nine deaths against 2.6 expected; $P = 0.001$), thyroid cancer (six against 1.8 expected; $P = 0.01$) and cancers of ill-defined and secondary sites (53 against 39.2 expected; $P = 0.02$). All of the deaths from cancer of the pleura were in radiation workers. The excess of thyroid cancer deaths was apparent in both radiation workers (four deaths *vs* 1.1 expected) and other workers (2 *vs* 0.7), but was significant ($P = 0.03$) only for the former group. Cancers of the pleura and of the thyroid were the only sites for which there was a significant excess of deaths among radiation workers. The excess of deaths from cancers of ill-defined and secondary sites was due mainly to an excess among non-radiation workers. Death rates from pleural and thyroid cancer were not available for Cumbria, but the excess of cancers of ill-defined and secondary sites was smaller, and not statistically significant, when the SMR was based on Cumbria rates. For all workers combined, there were significant deficits of cancers of the liver and gall bladder (two deaths *v* 11.1 expected; $P = 0.001$), larynx (2 *vs* 7.6; $P = 0.02$) and lung (SMR = 89; Cl = 79,100), though the last of these was not significant when comparison was made with Cumbria rates (SMR = 100; Cl = 88,112).

For no cancer site was the SMR among radiation workers significantly higher than that among other workers. In the total workforce, there were fewer deaths than expected from leukaemia (15 *vs* 22.9), and this deficit was significant among non-radiation workers (2 *vs* 6.7; $P = 0.04$) but not among radiation workers (13 *vs* 16.1; $P = 0.26$), though the difference between the two SMRs was not significant.

For analyses conducted of deaths from causes other than cancer, the findings were very similar to those previously reported (Smith & Douglas, 1986) and, therefore, we have not presented them in detail. Compared with England and Wales rates, there were significant excesses of deaths from mental disorders (26 *vs* 14.5; $P = 0.004$), ischaemic heart disease (1,124 *vs* 992.4; $P<0.001$) and ill-defined conditions (10 *vs* 4.5; $P = 0.02$) and significant deficits of tuberculosis (9 *vs* 21.7; $P = 0.002$), diseases of nervous and sense organs (28 *vs* 43.9; $P = 0.007$), pneumonia (96 *vs* 126.7; $P = 0.002$) and bronchitis (122 *vs* 158.6; $P = 0.001$). In no instance was the SMR for radiation workers significantly higher than those for other workers. The excess of ischaemic heart disease was not apparent when comparison was made with Cumbria mortality rates (SMR = 100; Cl = 94,106).

Mortality in relation to cumulative radiation dose among radiation workers is shown in Table IV for all causes, all malignant neoplasms and separately for those cancer sites for which there were five or more deaths. Analyses were conducted with radiation exposure 'lagged' by 2 or 10 years as well as with no lag. The last columns of the table show tests for trend in risk with cumulative radiation dose. The expected numbers of deaths shown in the body of the table were derived assuming no relationship between radiation dose (with no lag period) and risk of death.

There was no significant association between the risk of death and cumulative radiation dose (lagged by 0, 2 or 10 years) for all causes of death combined or for deaths from all malignant neoplasms. There were significant positive associations between cumulative radiation dose and death rates from cancers of ill-defined and secondary sites (with lag of 10 years; $P = 0.012$) and for leukaemia (no lag, $P = 0.004$; 2 year lag, $P = 0.009$). The latter association was slightly stronger if the one death from chronic lymphatic leukaemia was excluded. The positive association between deaths from myeloma (with a lag of 10 years) was not quite statistically significant, based on a simulated $P$-value ($P = 0.058$). There was a significant negative association between cumulative radiation dose (0 lag) and the risk of death from cancer of the kidney (simulated $P$-value 0.02, one-sided test). None of these findings were changed materially by excluding deaths (and person-years) within 5 years of first employment (a period when both the mortality rate and the accumulated radiation doses were relatively low).

In our previous analyses we reported on the association between cumulative radiation dose and some specific non-malignant causes of death: circulatory diseases, ischaemic heart disease, cerebrovascular disease, respiratory diseases, digestive diseases, genitourinary diseases and accidents and violence. The only significant effect found was a negative association between deaths from respiratory diseases and cumulative radiation dose (with no lag) (Smith & Douglas, 1986). In the updated analyses, this association was no longer statistically significant and nor were any of those for the causes of death listed above.

### *Cancer incidence*

Workers were included in the study of cancer registrations if they joined the Sellafield workforce after 1970 or if they joined the workforce prior to 1971 and were alive and not known to be resident outside of the UK on 1 January 1971. A total of 13,105 (92%) workers fulfilled these criteria and their cancer morbidity was studied from that date up to 1 January 1987. During this period cancers were registered in the national scheme for 653 members of the study population (Table V). The standardised registration rates (SRRs) for all cancers combined were lower than those of the general population of England and Wales (SRR = 90; $P = 0.003$; CI = 83,97) and of the general population of the Northern Region (SRR = 82; $P < 0.001$; CI = 76,88) (Table VI).

Table VII shows standardised registration ratios, based on both England and Wales and Northern region registration rates, for cancers of individual sites. In the total worker population there were significant excesses of cancer registrations for cancers of the oesophagus, pleura and ill-defined and secondary sites. The excesses were less marked and not significant when comparison was made with Northern Region rates. The excess of cancers of ill-defined and secondary sites was present for both radiation and other workers, but was greatest in the latter group. The excess of oesophageal cancer was in radiation workers only. There were significant deficits of cancers of liver and gall bladder, larynx and breast compared with England and Wales and Northern rates, and also for cancers of the mouth and pharynx, stomach, lung and bladder compared with Northern Region rates.

For only one cancer site, cancer of the prostate, was the SRR among radiation workers significantly greater than that among other workers. This was because of a deficit of these cancers among non-radiation workers. The only site for which the number of registrations was significantly in excess of expectation, among radiation workers, was for cancer of the oesophagus (22 registrations versus 12.9 expected; $P = 0.013$). There were significant deficits of registrations among radiation workers, compared with Northern rates, of cancers of the mouth and pharynx, stomach, larynx and lung.

The number of cancers of specific sites associated with different cumulative radiation doses is shown, for radiation workers, in Table VIII. Also shown are tests for the significance of the trend in the association between dose and risk. The only cancer site for which a significant association was found was for leukaemia (excluding chronic lymphatic leukaemia) with lags for the radiation doses of 0 or 2 years ($P = 0.04$ and $P = 0.03$, respectively, based on simulation tests).

### Discussion

The workers included in the study have been included in combined analyses of UK nuclear workers (Kendall et al., 1992a; Carpenter et al., 1994), but these gave only limited results for Sellafield workers specifically and neither included data on cancer incidence. The radiation doses that have been accumulated by workers at the Sellafield plant, since it opened in 1947, are higher, on average, than those experienced by workers at other nuclear facilities in the UK (Beral et al., 1988; Fraser et al., 1993) and in the USA (Gilbert et al., 1989; Wing et al., 1991). These workers are of special interest, therefore, with respect to their risk of radiation-induced cancers. We have reported previously on the mortality, up to 1984, of all those who worked at the plant at any time between 1947 and 1975 (Smith & Douglas, 1986) and we have now studied the mortality of this group up to 1989 and cancer incidence between 1971 and 1986. As the cohort has aged the overall death rate has increased and, by extending the follow-up by 5 years, the total number of deaths increased by 39% and the number from cancer by 46%. We are thus able to obtain a better estimate of the long-term risks associated with radiation exposure in the workforce.

The overall mortality rate in the cohort under study, during 1947–88, was close to that of the general population of England and Wales (SMR = 98) and 6% less than that of the population of Cumbria. The mortality rate from cancers of all kinds was similar to that of both England and Wales (SMR = 96) and of Cumbria (SMR = 100), and the overall cancer incidence rate was lower among Sellafield workers than those in the general population of England and Wales or Northern region (Table VII). The findings of mortality are very similar to those reported in our earlier paper (Smith & Douglas, 1986). In other studies of workers in nuclear plants, overall mortality rates have been found to be substantially lower than those of the general population (Beral et al., 1988; Wing et al., 1991; Fraser et al., 1993; Gilbert et al., 1993) and this has usually been attributed to the 'healthy worker' effect, often found in studies of occupational mortality, resulting from higher death rates in the general population among individuals with chronic sicknesses who either do not seek employment or who are not selected for employment. We commented at some length on the apparent absence of this effect among the Sellafield workers (Smith & Douglas, 1986) and concluded that it seemed unlikely that such an effect was masked owing to a deleterious effect of radiation exposure, the two principal reasons being that overall mortality rates were lower among radiation workers than among other workers and that there was little evidence of an association between accumulated radiation dose and death rates from all causes combined. These findings were replicated in the updated analyses (Tables III and IV).

Although there was no significant overall excess of deaths from cancer in the study population, there were significant excesses for cancers of three sites, cancer of the pleura, thyroid cancer and cancers of ill-defined and secondary cancers. The excess of ill-defined and secondary cancers was not significant compared with Cumbrian death rates and was largely attributable to an excess among non-radiation workers (Table III). These findings were more marked in our previous analysis, and in the extended follow-up period from 1984 to 1988 the number of deaths from this cause was similar to the number expected (19 against 16.1). However, previously we found only a weak association, among radiation workers, between the risk of death from this cause and accumulated radiation dose, whereas in the updated analysis this association was statistically significant, using a lag period of 10 years (Table IV). This finding is consistent with radiation being a cause of these cancers, but does not account for most of the excess being among those not monitored for radiation exposure. An excess of these cancers has not been found in other studies of nuclear workers and nor has an association with radiation dose (Beral et al., 1988; Fraser et al., 1993), and our findings are difficult to interpret. For 35 of the 53 deaths from this cause there was a cancer registration, but these did not help identify the site of the primary cancer in most cases, since for 28 the registration was also of an ill-defined or secondary cancer. The excess of these cancers was also evident in the analysis of the data on cancer incidence (Table VII), though there was no clear association with accumulated radiation dose (Table VIII).

Previously, we reported two deaths from cancer of the thyroid against 1.3 expected (Smith & Douglas, 1986). One death from this cause occurred in a worker who had not

**Table V** Sellafield workers included in the study of cancer incidence between 1 January 1971 and 1 January 1987

| | Men | Women | Total |
|---|---|---|---|
| Eligible for analysis[a] | 10,584 | 2,521 | 13,105 |
| Non-skin, malignant cancer before 1987 | 568 | 85 | 653 |
| Total years of follow-up | 148,879 | 37,154 | 186,033 |
| Average duration of follow-up | 14.1 | 14.7 | 14.2 |

[a]First employed at Sellafield after 1970 or first employed at Sellafield prior to 1971 and alive on 1 January 1971 and not lost to follow up or known to have emigrated before then.

**Table VI** Standardised registration ratios (SRRs) for cancers other than skin cancer, comparing rates in the study population with those of England and Wales (E&W) and of Northern Region

| | Men | Women | Total |
|---|---|---|---|
| No. of persons registered with a cancer | 568 | 85 | 653 |
| SRR (E&W) | 93* | 73*** | 90** |
| SRR (Northern Region) | 83*** | 71*** | 82*** |

*$P < 0.05$; **$P < 0.01$; ***$P < 0.001$.

been traced at the time of the last follow-up and three additional deaths occurred in the period 1984–88. The standardised mortality ratios were raised for both radiation and other workers, but only for the former group is the excess significant (Table III). The four radiation workers who died from this cause had accumulated doses of 8.1, 25.1, 38.7 and 909.1 mSv by the time of their deaths. There was no significant association between mortality rates and cumulative radiation dose (not shown in Table IV – the *z*-statistics using lags of 0, 2 and 10 years were 0.46, 0.50 and 0.78, respectively). A significant excess for thyroid cancer has not been reported in other studies of workers in specific nuclear plants (Beral *et al*., 1988; Fraser *et al*., 1993; Gilbert *et al*., 1993), though in the study of employees of the United Kingdom Atomic Energy Authority there were five deaths among non-radiation workers, against 2.1 expected. An excess of thyroid cancer was found in the first analysis of data from the National Register for Radiation Workers (Kendall *et al*., 1992*a*), but four of the nine deaths from thyroid cancer in that study were among Sellafield workers (Kendall *et al*., 1992*b*) and are included in our study. If these are removed the remaining excess is not significant (five against about three expected).

Cancer of the pleura was not analysed as a specific cause of death in our previous analyses, and national death rates from this cause were not available prior to the introduction of the eighth revision of the International Classification of Diseases and Causes of Death in 1968. Eight of the nine deaths from cancer of the pleura occurred between 1986 and 1988, and there were no deaths from this cause before 1978. Thus, the excess mortality is almost entirely in the extended follow-up period. All of the deaths were among radiation workers, but the expected number of deaths among other workers was small (Table III) and the rates in the two groups were not significantly different. There was little evidence of an association between accumulated radiation dose and the risk of death from this cause (Table IV). In addition to the nine deaths for which cancer of the pleura was coded as the underlying cause of death, there were four deaths for which mesothelioma was mentioned on the death certificate (two in radiation workers). Data were kindly supplied to us by the Health and Safety Executive from their Mesothelioma Register on the number of death certificates on which there was mention of mesothelioma for all deaths in England and Wales and Scotland between 1968 and 1991. Using age-, sex- and year-specific rates based on these numbers, we calculated the expected number of such deaths among Sellafield workers after 1967. The observed number, 13, was significantly greater than the number expected, 3.98 ($P<0.001$). There is substantial geographical variation in mesothelioma rates, and those in Cumbria and Northern Region are about twice the national average (Jones *et al*., 1988) (see also Table VII). However, rates in Cumberland, in which Sellafield is sited, are about half the national average (Swerdlow & dos Santos Silva, 1993). Exposure to asbestos is known to be a strong risk factor for mesothelioma. We do not know the extent of such exposure in the Sellafield plant or in other employments of those dying of mesothelioma, though some are known to have been so exposed (A. Slovak, personal communication). It is possible that radiation workers were more likely to be so exposed. Further investigation of this would seem warranted. The induction period for mesothelioma following asbestos exposure may be several decades, and it is of concern that most of the deaths from this cause occurred in the last 3 three years of follow-up. Continued monitoring for mesothelioma in the cohort will be important. An excess of cancer of the pleura among radiation workers was found in the combined analysis of mortality in three UK nuclear industry workforces, but there was not a significant excess if Sellafield workers were excluded (Carpenter *et al*., 1994).

The excess of cases of cancer of the oesophagus among radiation workers was significant only for cancer registrations (Table VII) and not for deaths (Table III). For neither deaths nor registrations was there a significant association with accumulated radiation dose (Tables IV and VIII).

In total we analysed data on deaths and registrations from cancers of over 30 sites, and it is to be expected that there will be some statistically significant findings by chance alone. There were no strong a priori reasons for supposing that any carcinogenic effect of radiation would be most marked for the sites discussed above. Leukaemia is more sensitive to radiation induction than are other cancers, but for both radiation and other workers the numbers of deaths and registrations from this cause were less than those expected, based on national or regional rates.

The significant deficits of cancers of the lung and larynx may be because the Sellafield workers smoked less than the general population. We have no data on this, but it should be noted that the rates of lung cancer in the worker population were similar to those of the population of Cumbria (Table III). We have no explanation for the significant deficit of deaths from liver cancer, which was also apparent in our earlier analysis (Smith & Douglas, 1986), or for the deficit of registrations of breast cancer among non-radiation workers (Table VII).

Radiation workers may differ from other workers and from the general population with respect to their risks of cancer, independently of any effect of radiation exposure, owing to differences in their socioeconomic characteristics and their exposure to other carcinogenic agents. The most informative, and probably least biased, analyses to assess specific carcinogenic effects of radiation are those based on comparison of the mortality rates of groups of radiation workers accumulating different doses of radiation. In these analyses a highly significant association was found between the risk of death from leukaemia and cumulative radiation dose. Studies of populations exposed to high radiation doses have shown that the shortest induction period for leukaemia, following radiation exposure, is about 2 years, compared with about 10 years for other cancers, and thus the analyses using a lag of 2 years are most relevant for leukaemia. In our earlier analysis we found a positive trend when leukaemia risk was related to radiation dose, which was most apparent using a 15 year lag period (Smith & Douglas, 1986). The more recent findings, with a 2 year lag giving the strongest association, are more consistent with those found in studies of populations exposed to high radiation doses.

The association between accumulated radiation dose and deaths from myeloma was significant in our earlier analyses using a lag period of 15 years. No more deaths from this cause occurred in the extended follow-up period and the original association remains, though now of marginal significance (*z*-statistic: 10 year lag 1.71, 15 year lag 1.79). Analysis of the mortality of workers at the Hanford nuclear facility in the USA has shown an association between deaths from myeloma and accumulated radiation dose (Tolley *et al*., 1983) but, when the cohort was followed for a longer period, the association was significant only using a lag period of 2 years and not with a lag of 10 years (Gilbert *et al*., 1993). No association was found in studies of the United Kingdom Atomic Energy Authority workers (Fraser *et al*., 1993) or the Atomic Weapons Establishment (Beral *et al*., 1988). The correlation found in the analysis of the National Registry of Radiation Workers (Kendall *et al*., 1992*a*) can be attributed to the inclusion of the Sellafield workers in that study (Kendall *et al*., 1992*b*).

A primary objective in studying the mortality of radiation workers who have been employed at the Sellafield plant has been to determine if any excess cancer mortality observed is consistent with the carcinogenic effects predicted from groups exposed to much higher doses of radiation than nuclear workers. In Table IX risk estimates are given, based on excess risk and excess relative risk models, together with estimates of risk derived from studies of the atomic bomb survivors. For leukaemia, radiation doses have been lagged by 2 years and, for other cancers, by 10 years. For cancers other than leukaemia the estimates of risk for radiation workers are consistent with those derived for adults from the atomic bomb survivors, but the confidence intervals include no effect and effects two or three times higher than those for

**Table IX** Risk estimates for radiation-induced deaths from leukaemias and all cancers other than leukaemia, using absolute and relative risk models

| *Lag period* | *Excess risk per $10^4$ person–years per Sv (90% confidence interval)* | *Excess relative risk per Sv (90% confidence interval)* |
|---|---|---|
| *Leukaemia (excluding chronic lymphatic)*[b] | | |
| 2 years | 2.47 (1.21, –)[a] | 13.92 (1.94, 70.52) |
| Atomic bomb survivors (adults)[c] | 3.9 | 3.8 |
| Males only | 5.0 | 3.7 |
| *All cancers except leukaemia* | | |
| 10 years | 5.60 (– 15.86, 27.15) | 0.11 (–0.43, 0.81) |
| Atomic bomb survivors (adults)[c] | 16.0 | 0.35 |
| Males only | 15.0 | 0.24 |

[a]Upper limits could not be estimated. [b]Excluded because there is no evidence that chronic lymphatic leukaemia is induced by radiation exposure. [c]Estimates of risk derived from A-bomb survivor data (UNSCEAR, 1988).

the atomic bomb survivors. For leukaemia the estimate based on the excess risk model is below that of the atomic bomb survivors, but the estimate based on the excess relative risk model is about four times higher, but with a confidence interval that extends from one-half to nearly 20 times the risk estimate based on the adult atomic bomb survivors. The majority of radiation workers at Sellafield were male and, therefore, risk estimates are also shown in Table IX for adult male atomic bomb survivors.

The International Commission on Radiological Protection has recommended that the risk estimates based on the atomic bomb survivors should be divided by 2 for populations exposed to low doses at low dose rates [dose and dose rate effectiveness factor (DDREF) of 2] (International Commission on Radiological Protection, 1991). This would have the effect of bringing the estimates based on the atomic bomb survivors closer to those derived for the Sellafield workers, with the exception of the excess relative risk model for leukaemia. More precise estimates of the carcinogenic effects of radiation exposure among workers in the nuclear industry will come from combining data for workers in other nuclear plants. Such studies have been conducted for workers in the UK (Kendall *et al.*, 1992*a*; Carpenter *et al.*, 1994), the USA (Gilbert *et al.*, 1994) and internationally (International Agency for Research on Cancer, 1994) and, taken together, provide little evidence that the estimates that form the basis of current radiation protection recommendations are appreciably in error. The risk estimate for leukaemia in the Sellafield workers, under the excess relative risk model, is higher than that found in other studies of workers in the nuclear industry. This may be due to chance or may relate to an increased risk due to other exposures in the plant which are more likely in those accumulating higher external radiation doses. It should be noted, however, that there is no evidence of an overall excess risk of leukaemia in the Sellafield workers compared with national or Cumbrian leukaemia rates (Table III).

This study was supported, in part, by a grant to the London School of Hygiene and Tropical Medicine by British Nuclear Fuels. We are grateful to the many people who have helped us with the study and, in particular, to the staffs of the NHS Central Registers, the medical statistics division of the Office of Population Censuses and Surveys and the national insurance records branch of the DSS. We thank the staff of British Nuclear Fuels with whom we liaised to assemble the data for analysis, especially Keith Binks, Andy Slovak and Sheila Jones.

### References

BERAL, V., FRASER, P., CARPENTER, L.M., BOOTH, M., BROWN, A. & ROSE, G. (1988). Mortality of employees of the Atomic Weapons Establishment, 1951–82. *Br. Med. J.*, **297**, 757–770.

CARPENTER, L., HIGGINS, C., DOUGLAS, A., FRASER, P., BERAL, V. & SMITH, P. (1994). Combined analysis of mortality in three UK nuclear industry workforces. 1946–88. *Radiat. Res.*, **138**, 224–238.

FRASER, P., CARPENTER, L., MACONOCHIE, N., HIGGINS, C., BOOTH, M. & BERAL, V. (1993). Cancer mortality and morbidity in employees of the United Kingdom Atomic Energy Authority, 1946–86. *Br. J. Cancer*, **67**, 615–624.

GARDNER, M.J., WINTER, P.D., TAYLOR, C.P. & ACHESON, E.D. (1983). *Atlas of Cancer Mortality in England and Wales*. Wiley: Chichester.

GARDNER, M.J., WINTER, P.D. & BARKER, D.J.P. (1984). *Atlas of Mortality from Selected Diseases in England and Wales, 1968–1978*: Wiley: Chichester.

GILBERT, E.S. (1989). Issues in analysing the effects of occupational exposure to low levels of radiation. *Statistics in Medicine*, **8**, 173–187.

GILBERT, E.S., PETERSEN, G.R. & BUCHANAN, J.A. (1989). Mortality of workers at the Hanford site: 1945–1981. *Health Physics*, **56**, 11–25.

GILBERT, E.S., OMOHUNDRO, E., BUCHANAN, J.A. & HOLTER, N.A. (1993). Mortality of workers at the Hanford site 1945–1986. *Health Phys.*, **64**, 577–590.

GILBERT, E.S., CRAGLE, D.L. & WIGGS, L.D. (1994). Updated analyses of combined mortality data for workers at the Hanford site, Oak Ridge National Laboratory, and Rocky Flats weapons plant. *Radiat. Res.*, **136**, 408–421.

HANLEY, J. & DOUGLAS, L. (1985). Fitting relationships between exposure and standardized mortality ratios. *J. Occup. Med.*, **27**, 555–560.

INTERNATIONAL AGENCY FOR RESEARCH ON CANCER (1994). Study Group on Cancer Risk among Nuclear Industry Workers. New estimates of cancer risk due to low doses of ionizing radiation: an international study. *Lancet* (in press).

INTERNATIONAL COMMISSION ON RADIOLOGICAL PROTECTION (1991). 1990 recommendations of the International Commission on Radiological Protection (ICRP Publication 60). *Ann. ICRP.*, **21**, 1–3.

JONES, R.D., SMITH, D.M. & THOMAS, P.G. (1988). Mesothelioma in Great Britain in 1968–1983. *Scand. J. Work Environ. Hlth.*, **14**, 145–152.

KENDALL, G.M., MUIRHEAD, C.R., MACGIBBON, B.H., O'HAGAN, J.A., CONQUEST, A.J., GOODILL, A.A., BUTLAND, B.K., FELL, T.P., JACKSON, D.A., WEBB, M.A., HAYLOCK, R.G.E., THOMAS, J.M. & SILK, T.J. (1992*a*). Mortality and occupational exposure to radiation: first analysis of the National Registry for Radiation Workers. *Br. Med. J.*, **304**, 220–225.

KENDALL, G.M., MUIRHEAD, C.R., MACGIBBON, B.H., O'HAGAN, J.A., CONQUEST, A.J., GOODHILL, A.A., BUTLAND, B.K., FELL, T.P., JACKSON, D.A., WEBB, M.A., HAYLOCK, R.G.E., THOMAS, J.M. & SILK, T.J. (1992*b*). *First Analysis of the National Registry for Radiation Workers: Occupational Exposure to Ionising Radiation and Mortality*, Publication NRPB-R251. National Radiological Protection Board: Chilton, Didcot.

LAND, C. (1980). Estimating cancer risks from low doses of ionizing radiation. *Science*, **209**, 1197–1203.

SMITH, P.G. & DOUGLAS, A.J. (1986). Mortality of workers at the Sellafield plant of the British Nuclear Fuels. *Br. Med. J.*, **293**, 845–854.

SWERDLOW, A. & DOS SANTOS SILVA, I. (1993). *Atlas of Cancer Incidence in England and Wales 1968–85*. Oxford University Press: Oxford.

TOLLEY, H.D., MARKS, S., BUCHANAN, J.A. & GILBERT, E.S. (1983). A further update of the analysis of mortality of workers in a nuclear facility. *Radiat. Res.*, **95**, 211–213.

UNSCEAR (1988). *Sources and Effects of Ionizing Radiation*, United Nations Scientific Committee on the Effects of Atomic Radiation: United Nations: New York.

WING, S., SHY, C.M., WOOD, J.L., WOLF, S., CRAGLE, D.L. & FROME, E.L. (1991). Mortality among workers at Oak Ridge National Laboratory. *JAMA*, **265**, 1397–1402.